# 

**Published:** 1859-04

**Authors:** 


*The American Medical Associntion.*—Our readers will recollect that the twelfth annual meeting of the American Medical Association, will take place the first Tuesday in May, at Louisville, Ky. There will, undoubtedly, be a full meeting, and the hospitality of the delightful city of Louisville will render this probably one of the most brilliant of the sessions of the Association. We insert the official announcement of the secretary.

The Twelfth Annual Meeting of this Association, will be held in Louisville, Ky., on Tuesday, May 3d, 1859. The secretaries of all societies, and other bodies entitled to representation in the Association, are requested to forward to the secretary, S. M. Bemiss, at Louisville, correct lists of their delegations so soon as they may be appointed. The Convention of Teachers, invoked by a resolution of the National Association, for the purpose of a general conference upou the best means of elevating the standard of medical education in this country, will meet in the same city, on Monday, 2d of May.

Medical Journals, throughout the United States, are requested to insert the above.

S. M. Bemiss,
Secretary American Medical Association.

---

*Researches on the Malignant Pustule of Man and Animals.*
By Dr. R. Virchow.

The author has ascertained by experiment that this disease may be propagated by inoculating, not only with the matter of the pustule, but with the blood of the diseased body, and that the time which elapses between inoculation and death varies between 44 and 66 hours, with one exception, in which a sheep died in 31 hours. He has also investigated with much care the character of the blood, and found that the white corpuscles are present in greater numbers, with some vibriones. These latter bodies were met with in living blood, and consequently they are not to be regarded as the products of decomposition.—*Archiv. für Pathologische Anatomie*, New Series, vol. i, 1857.

